# Interleukin-10 is not associated with obstructive sleep apnea hypopnea syndrome: A meta-analysis and meta-regression

**DOI:** 10.1097/MD.0000000000035036

**Published:** 2023-09-22

**Authors:** Xiaoyan Li, Lingyun Wu, Jie He, Qiuhua Sun

**Affiliations:** a School of Nursing, Zhejiang Chinese Medical University, Hangzhou, China; b Clinical Medical College of Chengdu Medical College, Chengdu, Sichuan, China; c Department of Pulmonary and Critical Care Medicine, The First Affiliated Hospital of Chengdu Medical College, Chengdu, China.

**Keywords:** bioinformatics, interleukin-10, meta-analysis, obstructive sleep apnea hypopnea syndrome, systematic review

## Abstract

**Background:**

This study was conducted to explore the potential relationship between interleukin-10 (IL-10) and obstructive sleep apnea hypopnea syndrome (OSAHS).

**Methods:**

All the related research articles published before October 2022 were retrieved through the online database (EMBASE, VIP, Wan Fang, Web of Science, PubMed, and CNKI). Stata 11.0 software was used to calculate the standard mean difference (SMD) of the continuous variable and 95% confidence interval (CI). Expression profiles GSE38792 and GSE135917 were acquired from Gene Expression Omnibus (GEO) database, respectively. The expression of IL-10 mRNA in subcutaneous adipose tissue and visceral adipose tissue of OSAHS patients and healthy subjects was extracted by R software to verify the difference in IL-10 between the 2 groups.

**Results:**

The IL-10 level in the plasma of people with and without OSAHS (STD Mean Difference (SMD) = −0.68, 95% CI = −1.58 to 0.21, *I*^2^ = 94.3%, *P* = .136) was the same. There was also no difference in IL-10 levels in serum between people with and without OSAHS (SMD = −0.12, 95% CI = −0.55 to 0.32, *I*^2^ = 94.4%, *P* = .591). In addition, the subjects were divided into different subgroups for meta-analysis according to race, body mass index, age, study type, and disease severity. Based on the outcomes, no notable difference was observed in the plasma/serum IL-10 level between the OSAHS subgroups and the control group. The results of bioinformatics analysis indicated that there was no significant difference in the expression of IL-10 mRNA in subcutaneous adipose tissue and visceral adipose tissue between patients with OSAHS and those in the control group.

**Conclusion:**

The current meta-analysis highlighted that IL-10 levels between patients with OSAHS and healthy people had no difference.

## 1. Introduction

Obstructive sleep apnea hypopnea syndrome (OSAHS) is a sleep-related respiratory disease caused by a variety of factors. It is characterized by consistent partial or complete collapse of the airway during sleep, resulting in insufficient ventilation in breathing (decreased during sleep) at night or apnea (the airflow is completely stopped during sleep).^[1]^ OSAHS is closely related to insulin resistance, obesity, metabolic syndrome, diabetes, and cardiovascular diseases.^[2]^ Based on the corresponding reports, the overall prevalence of OSAHS is 9% to 38% in healthy adults, of which moderate to severe OSAHS were predominant. The prevalence of OSAHS could be as high as 49.7% in men, while women have a level of 23.4%. OSAHS is also common in children with an estimated prevalence of approximately 5.7%.^[3–5]^ Furthermore, OSAHS is also related to weight; obesity is the most important risk factor among other factors of OSA. Forty percent of obese individuals have OSAHS, whereas 70% of the patients with OSAHS are obese.^[6]^

OSAHS is a severe threat to human health. Large-scale epidemiological and prospective studies have demonstrated that OSAHS increases the incidence of obesity, metabolic syndrome, and risk of cardiovascular complications.^[7]^ According to the literature, OSAHS is related to the high incidence of cancer that could be related to recurring night hypoxia and high carbonate levels attributed to OSAHS.^[8]^ Moreover, intermittent hypoxia caused by OSAHS can induce various peripheral blood cells to produce active oxygen, causing an upregulation of proactive factors and downregulation of anti-inflammatory factors.^[9]^ Repeated hypoxia and loss of oxygen attributed to OSAHS can trigger an anti-inflammatory reaction; thus, OSAHS is considered a low-degree chronic inflammatory disease.^[10]^ Classic anti-inflammatory factor levels, such as interleukin-10 (IL-10), have been observed to be downregulated in children and adult patients with OSAHS.^[11,12]^ Li et al^[13]^ also demonstrated that the down-regulation of IL-10 could promote the activation of the nuclear factor kappa B (NF-κB) pathway that further aggravates the inflammatory response. IL-10 is secreted by activated T cells (mainly Treg cells and Th2 cells), monocytes/macrophages, and mast cells that are multifunctional cytokines with extensive anti-inflammatory effects and can inhibit pro-inflammatory factors, such as IL-6, IL-8, and tumor necrosis factor (TNF)-α, etc., inhibiting the antigen presentation of macrophages and neutrophil aggregation.^[14]^ Decreased levels of IL-10 promote the secretion of inflammatory factors enhancing vascular inflammation that could result in OSAHS -related cardiovascular and cerebrovascular diseases.^[15]^ However, some study showed that serum IL-10 levels in OSAHS patients did not differ from controls. Any relationship between IL-10 concentration and OSAHS remains controversial.

A previous meta-analysis^[16]^ reported that plasma IL-10 levels could be a good marker for identifying or excluding adult OSAHS; this study was a diagnostic meta-analysis that neither used the standardized mean difference (SMD) to analyze the data nor conducted a subgroup analysis based on the race or body mass index (BMI). Furthermore, only 9 studies were included in the above-mentioned meta-analysis, and the sample sizes were relatively small. In order to address these limitations, we used SMD to conduct a comprehensive analysis of the IL-10 levels in patients with OSAHS based on 25 studies. We also tested the correlation between age, BMI, apnea–hypopnea index (AHI), ethnicity, study design and IL-10 based on meta-regression. Materials and Methods

## 2. Methods

The review process followed the guidelines mentioned in the preferred reporting item for systematic reviews and meta-analysis (PRISMA) statement.^[17]^ The meta-analysis was registered at the prospective register of systematic reviews (PROSPERO, https://www.crd.york.ac.uk/PROSPERO/; ID: CRD42021274507).

### 2.1. Retrieval strategy

We comprehensively searched EMBASE, VIP, Wanfang, Web of science, PubMed, and CNKI for articles published before October 27, 2022, without any restrictions. The following search terms and key words were applied to identify eligible publications: “Obstructive sleep apnea hypopnea syndrome” or “Obstructive sleep Apnea” or “Obstructive sleep Apnea syndrome” or “OSA” or “OSAHS” or “OSAS,” “expression,” “interleukin-10” OR IL-10.” The reference lists of the eligible studies were also manually screened to determine other relevant articles.

### 2.2. Qualification criteria

The inclusion criteria were as follows: Case-control, cohort, or cross-sectional studies without age, sex, or BMI restrictions to evaluate the relationship between the IL-10 levels and OSAHS, OSAHS was defined as adult AHI ≥ 5 times/h and child AHI ≥ 1 times/h, and OSAHS severity was defined using traditional definitions(AHI < 5, normal; AHI 5 to 14, mild OSAHS; AHI 15 to 29, moderate OSAHS; and AHI ≥ 30, severe OSAHS,^[18]^ OSAHS was diagnosed with the aid of polysomnography, and Sufficient data were reported to calculate the SMD and 95% confidence interval (CI) in patients with OSA and those in the controls. The exclusion criteria are as follows: Studies with irrelevant, insufficient data, or without clinical data, Review articles, case reports, comments, letters, editorials, etc., Absence of a control group, and Overlap of the reported data with other studies.

### 2.3. Literature selection

We independently searched the above databases according to the retrieval strategy, and read the title and abstract of the article. All the selected full-text pieces of literature that presumably met the conditions were obtained. Later, we studied and evaluated the summary and entire text in detail, and redefined the articles that could be included. We consulted another expert in case of a disagreement regarding the inclusion of certain points in the article.

### 2.4. Data extraction

We independently extracted the data of each study included in the meta-analysis. The basic information extracted by the meta-analysis included the first author, publication year, country, participant race, age, BMI, and AHI, as well as the SMD of IL-10 level in the tissue samples.

### 2.5. Quality assessment

We assessed the quality of the studies that were included using the Newcastle-Ottawa scale; the highest total score for each study was 9. It could examine publications about the study population (4 items, full score 4), exposure or outcome (3 items, overall score 3), and comparability (1 item, overall score 2). Total respective scores of 7 to 9, 4 to 6, and 0 to 3 were considered high, medium, and low-quality studies.^[19]^

### 2.6. Bioinformatics

Data sets required for bioinformatics analysis from Gene Expression Omnibus database (GEO) (https://www.ncbi.nlm.nih.gov/geo/). GSE135917^[20]^ and GSE38792^[21]^ microarray datasets had the same gene annotation platform (HuGene-1_0-st; Affymetrix Human Gene 1.0 ST). GSE135917 dataset had 18 samples of subcutaneous adipose tissue along with normal controls (n = 8) and untreated OSAHS patients (n = 10). GSE38792 dataset had 18 samples of visceral adipose tissue along with normal controls (n = 8) and untreated OSAHS patients (n = 10). Table S1, Supplemental Digital Content, http://links.lww.com/MD/J823 showed the clinical data and basic information related to the patients in both datasets.

The “GEOquery” software package was employed to get the original data that was analyzed using the oligo software package in R software (version 4.1.0). The probe name was changed to the gene name following the manufacturer-provided annotation file. Probes without corresponding gene names were deleted. The “affy” software package in R software was used to preprocess and normalize the chip dataset. The “select” function in R software was used to extract the expression of IL-10 mRNA in each sample of the above 2 datasets.

### 2.7. Ethical review

Ethical approval and patient consent were not required because the meta-analysis and bioinformatic analyses were based on published research and public database.

### 2.8. Statistical analysis

The following data were analyzed. SMD and 95% CI were calculated by Stata 11.0 (Stata Corporation, College Station, TX), and the significance of the combined SMD was evaluated by the Z-test. Heterogeneity was assessed using Cochran Q and *I*^2^ indicators in all the studies, with scores ranging from 0% to 100%. In addition, significant heterogeneity was observed in the case of *P* heterogeneity with *P*_heterogeneity_ < 0.1 and *I*^2^ > 50%. In this case, the random effect model was analyzed to evaluate the combined SMD and 95% CI values. We also use the fixed-effect model for analysis. The sources of heterogeneity were estimated by meta-regression and sensitivity analysis, and subgroup analysis was performed according to race, mean AHI, mean BMI, age, study design; The publication bias was visually evaluated by funnel diagram asymmetry, and the test results of Egger’s and Begg’s were analyzed by Stata 11.0 software. Some studies reported values between the median and quartile, which were converted to mean and standard deviation.^[22]^ A non-parametric Wilcoxon rank sum test was used for the comparison of IL-10 mRNA levels between OSAHS fat tissues and normal fat tissues. The statistical analyses and visualization were performed by GraphPad Prism 8 (https://www.graphpad.com/scientific-software/prism/).

## 3. Results

### 3.1. Options for inclusion in the study

**Figure 1.**
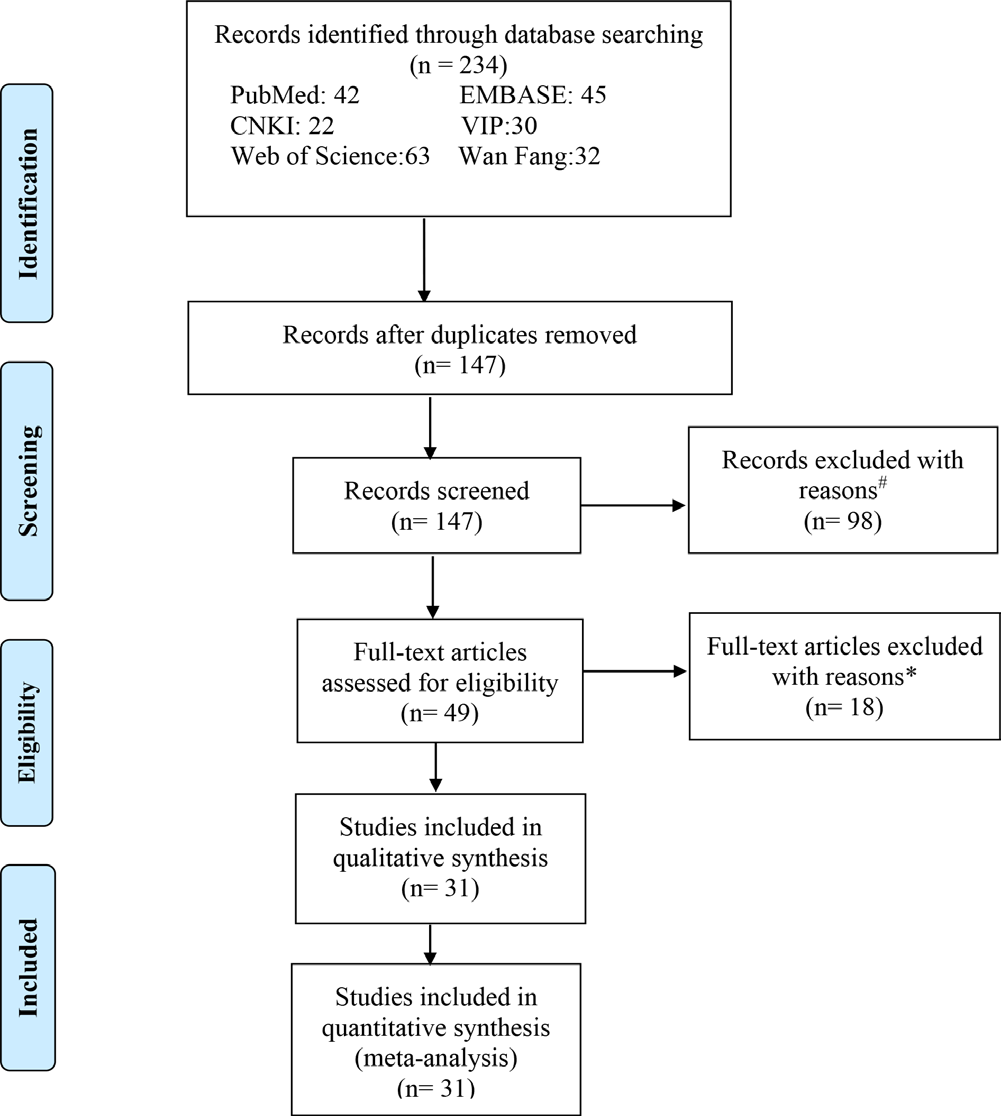
Flow diagram indicating the literature selection process and the results based on the preferred reporting items for the meta-analysis.

The retrieval strategy is shown in Figure 1. A total of 234 studies were identified; since 87 articles were repeated, they were deleted. The titles and abstracts of the remaining 147 articles were screened. Of these, 98 articles were considered insignificant and were therefore excluded. The remaining 49 articles were screened, and 18 articles were excluded for various reasons, such as lack of a control group, lack of data, studies published in languages other than English or Chinese, unclear measurement units, case reports, and reviews. Finally, 25 papers^[11,12,23–45]^ comprising 31 studies were included in the current meta-analysis, as showed in Figure 1.

### 3.2. General characteristics of the included studies

Tables 1 and 2 listed the general characteristics of the included studies. A total of 25 articles involving 31studies were included in this meta-analysis. These studies were published from 2002 to 2022. Among these studies, 11 studies^[11,24–26,29,30,32,36,41–45]^ reported the relationship between plasma IL-10 levels and OSAHS, A study reported differences in IL-10 levels in tonsil tissue between OSAHS patients and non-OSHAS patient,^[22]^ and 19 studies^[12,23,27,28,31,33–35,37–40]^ reported the relationship between the serum IL-10 levels and OSA. The determination methods of IL-10 varied across the studies; however, most of them use enzyme-linked immunosorbent assay (ELISA).

**Table 1:** Characteristics of included studies.

Author	Year	Country	Ethnicity	Case/Control (n)	Sample source	Assay approach	NOS	Study design	PSG type
Alberti A et al	2003	Italy	Caucasian	18/20	plasma	ELISA	8	Case-control	I
Bhatt SP et al	2021	India	Asian	190/57	Serum	ELISA	9	Cross-sectional	I
Leon-Cabrera S et al	2015	Mexico	Latino	10/29	Serum	ELISA	8	Case-control	I
Chen VG et al	2018	Brazil	Latino	17/17	Tonsil tissue	ELISA	6	Cross-sectional	I
Dalesio NM et al (Obese and OSAS)	2020	America	Caucasian	8/18	Plasma	Mass spectrometry	8	Case-control	I
Dalesio NM et al (OSAS Only)	2020	America	Caucasian	7/18	Plasma	Mass spectrometry	8	Case-control	I
Dyugovskaya L et al	2002	Israel	Caucasian	8/6	Serum	Flow	8	Case-control	I
						cytometer			
Galati D et al	2020	Italy	Caucasian	45/30	Serum	Flow	6	Cohort	I
						cytometer			
Gozal D et al	2008	America	Caucasian	20/20	Plasma	ELISA	8	Case-control	I
Hirsch D et al	2018	Australia	Caucasian	23/16	Serum	A multiplex bead-based	7	Cross-sectional	I
						assay			
Huang YS et al	2020	China	Asian	55/32	Serum	ELISA	8	Case-control	I
Jiang H et al(Mild)	2015	China	Asian	45/94	Serum	ELISA	7	Case-control	I
Jiang H et al(Moderate)	2015	China	Asian	44/94	Serum	ELISA	7	Case-control	I
Jiang H et al(Severe)	2015	China	Asian	46/94	Serum	ELISA	7	Case-control	I
Ke D et al	2019	Canada	Caucasian	10/5	Plasma	ELISA	8	Case-control	I
Alonso-Fernandez et al	2021	Spain	Caucasian	22/11	Plasma	ELISA	7	Case-control	I
Serednytskyy O et al	2022	Spain	Caucasian	17/34	Plasma	ELISA	7	Case-control	I
Li Y et al(Serum Mild)	2009	China	Asian	22/22	Serum	ELISA	8	Cross-sectional	I
Li Y et al(Serum Moderate)	2009	China	Asian	22/22	Serum	ELISA	8	Cross-sectional	I
Li Y et al(Serum Severe)	2009	China	Asian	24/22	Serum	ELISA	8	Cross-sectional	I
Niżankowska-Jędrzejczyk A et al	2014	Poland	Caucasian	22/16	Serum	ELISA	6	Case-control	I
Qian X et al	2012	China	Asian	30/40	Serum	ELISA	8	Case-control	I
Rogers VE et al	2018	America	Caucasian	20/7	Serum	ELISA	7	Cross-sectional	I
Ryan S et al(Mild to Moderate)	2006	Poland	Caucasian	35/30	Serum	ELISA	9	Cohort	I
Ryan S et al(Severe)	2006	Poland	Caucasian	31/30	Serum	ELISA	9	Cohort	I
Sahlman J et al	2010	Finland	Caucasian	84/40	Plasma	ELISA	7	Cross-sectional	I
Silva WA et al	2021	Brazil	Latino	188/520	Serum	ELISA	8	Cross-sectional	I
Su MS et al	2017	China	Asian	42/48	Plasma	ELISA	7	Case-control	I
Sarinc Ulasli S et al	2015	Turkey	Caucasian	28/20	Serum	ELISA	8	Cross-sectional	I
Wang W et al	2021	China	Asian	22/20	Plasma	ELISA	7	Case-control	I
Zhang Z et al	2017	China	Asian	50/52	Serum	Flow cytometry	6	Case-control	I

ELISA = Enzyme linked immunosorbent assay, NA = not available, NOS = Newcastle-Ottawa scale, PSG = polysomnography.

**Table 2:** Participants’ characteristics of included studies.

Author	IL-10 (Mean ± SD)		BMI (Mean ± SD)		Age (Mean ± SD)		AHI (Mean ± SD)	
	Case	Control	Case	Control	Case	Control	Case	Control
Alberti A et al	3.5 ± 2.7	5.4 ± 1.8	26.5 ± 2.2	22.1 ± 3.4	52.7 ± 12.0	51.3 ± 13.2	18.2 ± 15.1	<5
Bhatt SP et al	2.62 ± 0.39	2.1 ± 0.28	27.1 ± 6.5	27.4 ± 2.8	10.7 ± 3.1	11.8 ± 2.6	13.3 ± 6.5	1.88 ± 1.11
Leon-Cabrera S et al	74.4 ± 17	113.2 ± 12	45.2 ± 8.4	23.6 ± 2.1	37.2 ± 11.4	43.4 ± 11.5	NA	NA
Chen VG et al	13 ± 11.77	10.69 ± 12.95	NA	NA	NA	NA	NA	NA
Dalesio NM et al (Obese and OSAS)	1 ± 0.7	0.46 ± 0.16	NA	NA	7 ± 2.5	8.3 ± 2.12	NA	NA
Dalesio NM et al (OSAS Only)	0.59 ± 0.17	0.46 ± 0.16	NA	NA	6.0 ± 1.8	8.3 ± 2.12	NA	NA
Dyugovskaya L et al	6.1 ± 4.8	14.7 ± 4.9	29.36 ± 5.5	27.3 ± 3.4	53.5 ± 12.9	53.2 ± 10.4	NA	NA
Galati D et al	2.44 ± 0.91	1.1 ± 0.68	28 ± 2.2	26.3 ± 1.8	53.9 ± 11.6	55 ± 5.8	43.5 ± 38.5	<5
Gozal D et al	195.2 ± 33.6	458.5 ± 102.2	17.3 ± 0.6	17.1 ± 0.5	6.5 ± 0.6	6.4 ± 0.7	13.3 ± 2.8	0.0 ± 0.0
Hirsch D et al	2.08 ± 1.3	1.83 ± 0.77	NA	NA	10.0 ± 1.7	10.7 ± 1.2	NA	NA
Huang YS et al	2.74 ± 2.94	2.1 ± 1.41	16.8 ± 4.0	17.44 ± 30	7.6 ± 2.6	7.0 ± 0.6	15.7 ± 22.6	0.46 ± 0.28
Jiang H et al (Mild)	6.68 ± 4.74	8.76 ± 5.25	NA	27.5 ± 2.58	NA	47.2 ± 13.5	11.52 ± 3.9	1.6 ± 1.6
Jiang H et al (Moderate)	5.77 ± 4.06	8.76 ± 5.25	NA	27.5 ± 2.58	NA	47.2 ± 13.5	21.06 ± 8.64	1.6 ± 1.6
Jiang H et al (Severe)	4.87 ± 3.84	8.76 ± 5.25	NA	27.5 ± 2.58	NA	47.2 ± 13.5	35.1 ± 24. 7	1.6 ± 1.6
Ke D et al	102.28 ± 115.08	257.15 ± 100.02	15.4 ± 0.6	16.5 ± 1.0	2.7 ± 0.4	4.0 ± 0.6	NA	NA
Li Y et al (Serum Mild)	46.7 ± 4.6	50.1 ± 6.9	25.7 ± 4.2	23.3 ± 2.0	48 ± 12	43.0 ± 93.0	14.1 ± 3.5	2.9 ± 1.3
Li Y et al (Serum Moderate)	35.7 ± 5.3	50.1 ± 6.9	28.8 ± 5.3	23.3 ± 2.0	44 ± 13	43.0 ± 93.0	29.7 ± 5.5	2.9 ± 1.3
Li Y et al (Serum Severe)	24.6 ± 5.1	50.1 ± 6.9	28.67 ± 4.2	23.3 ± 2.0	44 ± 8	43.0 ± 93.0	70.1 ± 18.1	2.9 ± 1.3
Niżankowska-Jędrzejczyk A et al	5.45 ± 4.02	3.97 ± 1.37	30.1 ± 2.7	28.0 ± 3.3	52.5 ± 8.3	54.0 ± 2.0	23.6 ± 12.3	2.26 ± 1.97
Qian X et al	26.1 ± 20.98	38.6 ± 10.6	29.4 ± 2.1	29.4 ± 2.1	45.0 ± 9.0	46.3 ± 8.1	NA	NA
Rogers VE et al	1.7 ± 1.06	2.03 ± 1.76	NA	NA	NA	NA	13.1 ± 9.8	0.8 ± 0.3
Ryan S et al (Mild to Moderate)	1.19 ± 0.42	1.19 ± 0.41	32.9 ± 6.03	30.7 ± 3.1	42.0 ± 8.0	41.0 ± 8.0	15.9 ± 7.7	1.2 ± 1.0
Ryan S et al (Severe)	1.14 ± 0.42	1.19 ± 0.41	32.1 ± 3.5	30.7 ± 3.1	43.0 ± 9.0	41.0 ± 8.0	56.6 ± 20.9	1.2 ± 1.0
Sahlman J et al	1.28 ± 2.34	0.7 ± 1.51	32.5 ± 3.3	31.5 ± 3.5	50.4 ± 9.3	45.6 ± 11.5	9.6 ± 2.9	1.9 ± 1.4
Silva WA et al	2.59 ± 0.37	2.51 ± 0.52	28.7 ± 4.0	25.1 ± 3.7	47 ± 6	45 ± 5	27.0 ± 13.1	5.4 ± 5.1
Su MS et al	261.7 ± 18.5	338.2 ± 25.2	NA	NA	NA	NA	8.68 ± 4.56	2.40 ± 2.75
Sarinc Ulasli S et al	34.7 ± 12.4	32.5 ± 14	32.6 ± 4.4	30.4 ± 8	53.7 ± 12.7	45.3 ± 14	34.08 ± 52.7	2.3 ± 2.9
Wang W et al	5.51 ± 1.53	5.7 ± 1.2	21.6 ± 2.2	18.9 ± 1.7	6.58 ± 0.61	5.69 ± 0.78	33.4 ± 5.6	1.1 ± 0.2
Zhang Z et al	3.45 ± 0.19	3.01 ± 0.17	NA	NA	NA	NA	NA	NA
Alonso-Fernandez et al	1.08 ± 0.19	0.95 ± 0.13	27.2 ± 2.4	26.6 ± 3.2	33.9 ± 5	35.6 ± 3.1	7.79 ± 3.48	0.37 ± 0.49
Serednytskyy O et al	0.64 ± 0.59	0.64 ± 0.49	28.8 ± 6.06	26.04 ± 4.57	37.64 ± 4.04	35.35 ± 5.42	8.94 ± 5.90	0.5 ± 0.54

AHI = apnea–hypopnea index, BMI = body mass index.

### 3.3. Quality assessment

The quality assessment results of the included studies are shown in Table 1. Among the included studies, 27 were of high quality and the other 4 were of medium quality.

### 3.4. Meta-analysis

#### 3.4.1. Comparison of plasma IL-10 concentrations between OSAHS patients and control group.

**Figure 2.**
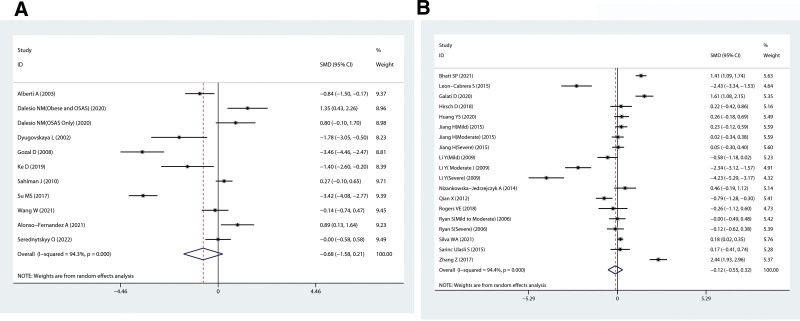
Standard mean difference (SMD) forest plot and its 95% confidence intervals (CI) for the interleukin-10 (IL-10) levels in the obstructive sleep apnea (OSAHS) group with the control group in the meta-analysis. A: plasma of IL-10, B: serum of IL-10.

The outcomes of a pooled analysis of plasma IL-10 concentrations in patients are illustrated in Figure 2A. The results indicated that no difference in the plasma levels of IL-10 was detected between OSAHS patients and controls (SMD = −0.68, 95% CI = −1.58 to 0.21, *P* = .136). The random-effects model was selected for investigation as a result of the high heterogeneity (*I*^2^ = 94.3%), as showed in Figure 2A.

#### 3.4.2. Comparison of serum IL-10 concentration among OSAHS patients and control group.

The correlation of serum IL-10 concentrations in patients with OSAHS with those in the control group was investigated in our meta-analysis, which included 19 eligible observational studies. Serum levels of IL-10 were no significant differences between the 2 groups (SMD = −0.12, 95% CI = −0.55 to 0.32, *P* = .591). For further investigation, the random-effects model was selected as a result of the increased heterogeneity (*I*^2^ = 94.4%, Fig. 2B).

#### 3.4.3. Sensitivity analysis.

**Figure 3.**
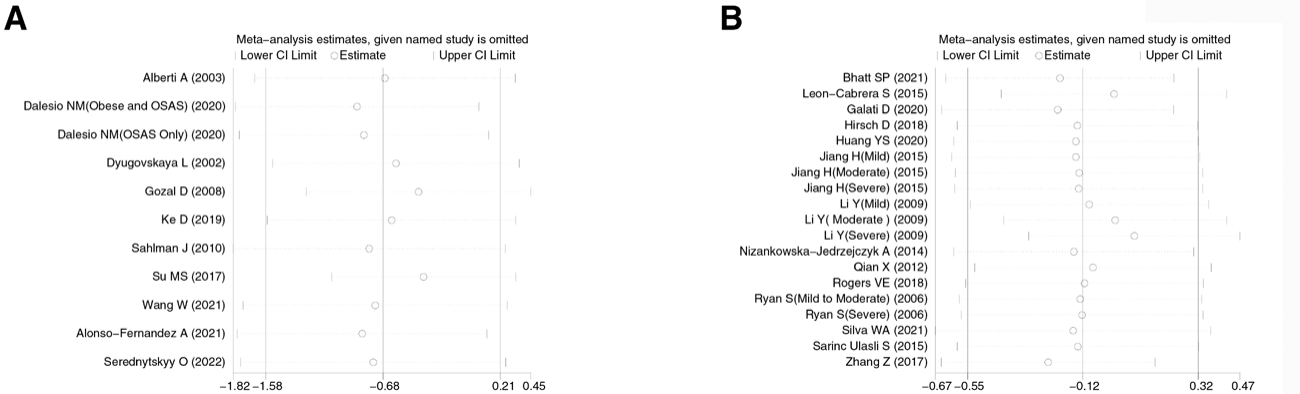
Sensitivity analysis of studies on IL-10 levels in patients with OSAHS versus controls. A: plasma of IL-10, B: serum of IL-10. OSAHS = obstructive sleep apnea hypopnea syndrome.

According to the literature on IL-10 expression levels in plasma and serum of patients with OSAHS, no studies were found showing an impact on the results after excluding each study in turn. Sensitivity analysis showed that the meta-analysis was stable (Fig. 3A and B).

#### 3.4.4. Comparison of palatine tonsils of IL-10 concentration among OSAHS patients and control group.

Chen et al^[25]^ collected tonsillar tissue samples from 34 children who underwent tonsillectomy (including 17 children with OSAHS and 17 children without OSAHS) and detected the expression of IL-10 and other inflammatory factors in the tissues using enzyme linked immunosorbent assay (ELISA). Chen et al^[25]^ found that the expression of IL-10 cytokines in tonsils of children with OSAHS was significantly increased (OSAHS group vs control group: 13.08 ± 11.77 pg/mL vs 10.69 ± 12.95 pg/mL, *P* = .04).

### 3.5. Subgroup analysis of plasma IL-10 concentration

#### 3.5.1. Age.

Plasma IL-10 levels in children with and without OSAHS were studied in 4 investigations. According to the findings, there was no major variation in plasma IL-10 levels between children with OSAHS and the control group (SMD = −1.05, 95% CI = −2.74 to 0.65, *P* = .226). Four studies compared plasma IL-10 concentrations in adults with and without OSAHS, indicating plasma IL-10 levels did not differ between the 2 groups (SMD = −0.17, 95% CI = −0.84 to 0.49, *P* = .613, Table 3).

**Table 3:** Subgroup analyses of the association between the interleukin-10 (IL-10) levels and OSAHS in the meta-analysis.

Subgroup analysis of plasma levels (n)	SMD (95% CI), *P* value, *I*^2^ (%), *P*_h_	Subgroup analysis of serum levels (n)	SMD (95% CI), *P* value, *I*^2^ (%), *P*_h_
Overall (11)		Overall (19)	
Ethnicity		Ethnicity	
Caucasian (9)	−0.41 (−1.21, 0.40), 0.320, 90.4%, <.001	Caucasian (7)	0.31 (−0.18, 0.81), 0.214, 96.5%, <.001
Asian (2)	−1.78 (−5.00, 1.44), 0.279, 98.1%, <.0001	Asian (10)	−0.28 (−1.05, 0.48), 0.471, 96.8%, <.001
Latino		Latino (2)	−1.09 (−3.65, 1.48), 0.407, 79.8%, <.001
BMI		BMI	
Mean BMI ≥ 30 (3)	−0.61 (−3.24, 2.02), 0.650, 98.2%, <.001	Mean BMI ≥ 30 (5)	−0.12 (−0.55, 0.32), 0.389, 95.5%, <.001
Mean BMI<30 (8)	−0.69 (−1.56, 0.18), 0.121, 89.3%, <.0001	Mean BMI<30 (14)	−0.05 (−0.57, 0.48), 0.854, 86.3%, <.001
Degree of severity		Degree of severity	
Mean AHI ≥ 30 (2)	−0.68 (−1.58, 0.21), 0.290, 95.3%, <.001	Mean AHI ≥ 30 (7)	−0.71 (−1.72, 0.30), 0.171, 95.6%, <.001
Mean AHI<30 (9)	−0.67 (−1.75, 0.41), 0.221, 70.4%, <.001	Mean AHI<30 (12)	0.18 (−0.29, 0.66), 0.447, 93.6%, <.001
Age		Age	
Adult (5)	−0.17 (−0.84, 0.49) , 0.613, 80.7%, <.001	Adult (14)	−0.44 (−0.89, 0.001), 0.050, 92.9%, <.001
Nonage (6)	−0.68 (−1.58, 0.21), 0.226, 96.0%, <.001	Nonage (5)	0.85 (−0.03, 1.73), 0.059, 93.4%, <.001
Design		Design	
Cross-sectional study (1)	NA	Cross-sectional study (8)	−0.60 (−1.42, 0.23), 0.155, 95.9%, <.001
Case-control study (10)	−0.79 (−1.82, 0.25), 0.138, 94.2%, <.001	Case-control study (8)	0.07 (−0.61, 0.75), 0.838, 94.3%, <.001
Cohort study	NA	Cohort study (3)	0.49 (−0.58, 1.56), 0.365, 94.4%, <.001

BMI = body mass index, OSAHS = obstructive sleep apnea hypopnea syndrome, SMD = positive likelihood ratio.

#### 3.5.2. BMI.

We performed subgroup analysis depending on whether the mean BMI was greater than 30, which was reported in all the included studies. Three studies reported plasma IL-10 concentrations in patients whose mean BMI was over 30, suggesting that plasma IL-10 concentrations in the OSAHS group were not lower or greater in comparison with those in the healthy control group (SMD = −0.61, 95% CI = −3.24 to 2.02, *P* = .650). According to 8 studies, the plasma IL-10concentrations in patients with an average BMI lower than 30 had no major variation between the OSAHS and the subjects of the control groups (SMD = −0.69, 95% CI = −1.56 to 0.18, *P* = .121, Table 3).

#### 3.5.3. Severity of OSAHS.

A subgroup meta-analysis of OSAHS severity was also undertaken. Plasma IL-10 levels were measured in 2 studies with an average AHI ≥ 30 and 9 studies reported plasma IL-10 levels in patients with an average AHI < 30. The outcomes revealed that regardless of whether the mean AHI of patients was greater than 30, there was no major variation in plasma IL-10levels between OSAHS patients and the control group (SMD = −0.66, 95% CI = −1.88 to 0.56, *P* = .290; SMD = −0.67, 95% CI = −1.75 to 0.41, *P* = .221, Table 3).

#### 3.5.4. Ethnicity.

The subgroup study of plasma IL-10 concentrations in OSAHS patients belonging to different ethnicities is compiled in Table 3. Caucasian and Asian populations are shown as the major subgroups. In the Caucasian population, there was no major variation in plasma IL-10 concentration between the OSAHS and control groups (SMD = −0.41, 95% CI = −0.121 to 0.40, *P* = .320). In the Asian population group, at the plasma IL-10 levels, similar result was observed (SMD = −1.79, 95% CI = −5.00 to 1.44, *P* = .279, Table 3).

#### 3.5.5. Research design type.

We conducted a matching subgroup analysis because the differences in design types included in the studies might alter the heterogeneity of the results. No major variation was seen in plasma IL-10 concentration between OSAHS patients and the control group (SMD = −0.44, 95% CI = −0.89 to 0.00, *P* = .05) according to a subgroup analysis of case-control studies. One study was a cross-sectional study and the majority were case-control studies (Table 3).

### 3.6. Subgroup analysis of serum IL-10 concentrations

#### 3.6.1. Age.

The serum IL-10 levels of OSAHS children and healthy children were evaluated in 5 investigations. The combined results revealed that there was no major variation in serum IL-10 levels between children with OSAHS and the control group (SMD = 0.85, 95% CI = −0.03 to 1.73, *P* = .059). Fourteen studies compared serum IL-10 concentrations in adults with and without OSAHS, indicating serum IL-10 levels did not differ between the 2 groups (SMD = −0.44, 95% CI = −0.89 to 0.00, *P* = .05, Table 3).

#### 3.6.2. BMI.

Based on BMI, we conducted a subgroup meta-analysis of 19 studies. Five studies were based on serum IL-10 concentrations of patients with an average BMI ≥ 30, and the outcomes of the analysis suggested that serum IL-10 levels did not differ from those of healthy controls in patients with OSAHS (SMD = −0.32, 95% CI = −1.04 to 0.41, *P* = .389). Fourteen publications gave us data on serum IL-10 concentration in patients with an average BMI < 30, and the outcomes of the analysis suggested that serum IL-10 levels did not differ from those of healthy controls in patients with OSAHS (SMD= = −0.32, 95% CI = −1.04 to 0.41, *P* = .389, Table 3) nether.

#### 3.6.3. OSAHS of severity.

Many studies have found a close link between serum IL-10 concentration to the AHI value in patients with OSAHS. In the current report, a comparison of serum IL-10 concentrations between OSAHS patients with an average AHI ≥ 30 and the control group were carried out in 7 studies, and the findings suggested that serum IL-10 levels in these patients were not significant different as compared to those in the control group (SMD = −0.71, 95% CI = −1.72 to 0.30, *P* = .171). Twelve studies were based on a comparison of the serum IL-10 concentrations between OSAHS patients with an average AHI < 30 and the control group, and the outcomes indicated that serum IL-10 concentrations in these patients were not significant different when compared with subjects of the control group (SMD = 0.18, 95% CI = −0.29 to 0.66, *P* = .447, Table 3).

#### 3.6.4. Ethnicity.

There were 19 reports from Caucasians, Asians, and Latin Americans included in the study. The serum IL-10 concentrations of OSAHS patients in these 3 groups exhibited no significant differences between OSAHS group and the control group, according to subgroup analysis based on different demographics and ethnicities (see Table 3).

#### 3.6.5. Research design type.

In cross-sectional studies, blood IL-10 concentrations in patients with OSAHS presented with comparable levels as those in control group (SMD = −0.60, 95% CI = −1.42 to 0.22, *P* = .155). Similarly, serum IL-10 concentrations in patients with OSAHS were not statistically different between OSAHS patients and controls in case-control and cohort studies (SMD = 0.07, 95% CI = −0.61 to 0.75, *P* = .838; SMD = 0.49, 95% CI = −0.57 to 1.56, *P* = .591, Table 3).

### 3.7. Meta-regression

All the included studies had an *I*^2^ value of 94.3% in plasma IL-10 and an *I*^2^ value of 94.4% in serum IL-10, indicating a high level of heterogeneity. As a result, we explored the possible sources of this heterogeneity using meta-regression. Table 4 demonstrates the meta-regression of serum/plasma IL-10 concentration. Unfortunately, the potential sources of heterogeneity were explored through meta-regression analysis; however, they were not determined.

**Table 4:** Meta-regression analysis of variables predicting serum and plasma levels of IL-10.

Variables	*R*	Adjusted *R*^2^	*P*
Age			
Serum	−1.339	0.139	.079
Plasma	0.786	−0.040	.461
BMI			
Serum	−0.274	−0.057	.733
Plasma	0.096	−0.121	.936
Severity			
Serum	−0.873	0.029	.223
Plasma	−0.066	−0.118	.963
Ethnicity			
Serum	−0.281	−0.032	.454
Plasma	−0.672	0.019	.317
Design			
Serum	0.031	−0.067	.950
Plasma	1.062	−0.077	.560

BMI = body mass index.

### 3.8. Publication bias

**Figure 4.**
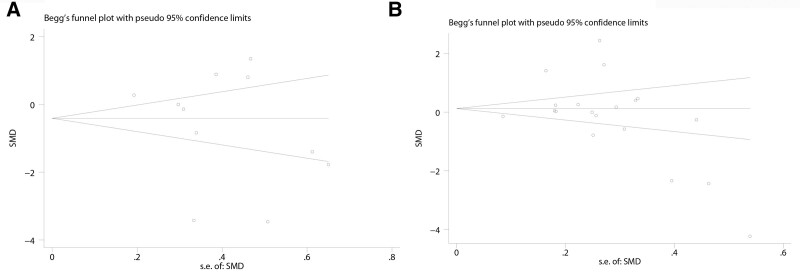
Funnel plot was employed to assess the publication bias among the included studies concerning the association of the interleukin-10 (IL-10) levels with OSAHS. A: plasma of IL-10, B: serum of IL-10. OSAHS = obstructive sleep apnea hypopnea syndrome.

Funnel plots were used to investigate the probability of publication bias in research investigating the link between IL-10 concentration and OSAHS, and our funnel plots appear to be symmetric. Begg’s and Egger’s tests (plasma: Begg’s (*P* = .276), Egger’s (*t* = −0.93, *P* = .379); serum: Begg’s (*P* = .162), Egger’s (*t* = −0.77, *P* = .453)) could not report any publication bias in the studies of patients with OSAHS (Fig. 4A and B).

### 3.9. Bioinformatic results

**Figure 5.**
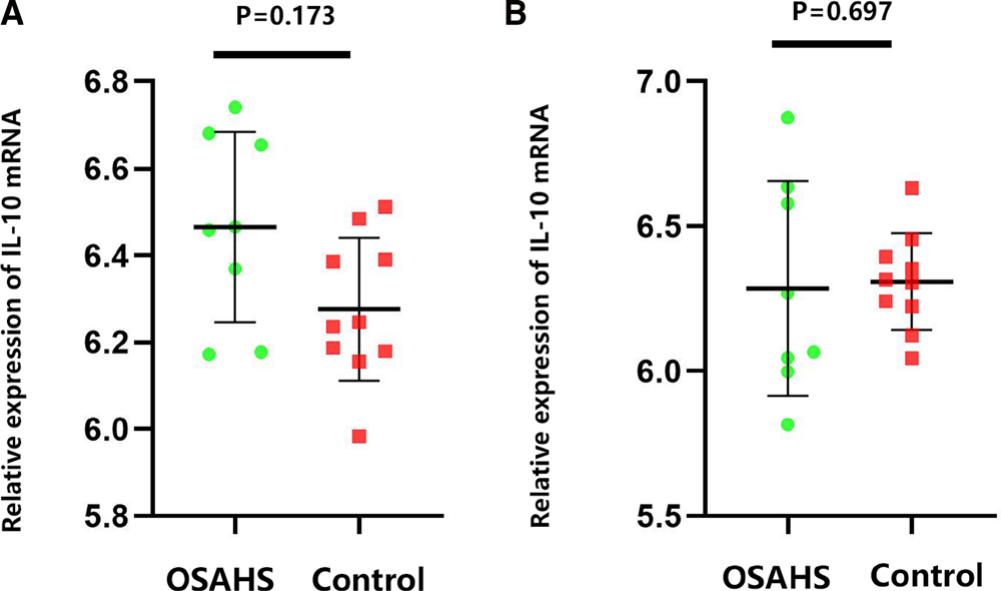
Expression of IL-10 in adipose tissues. A: gene expression of IL-10 in the subcutaneous adipose tissues (GSE135917). B: gene expression of IL-10 in the visceral adipose tissues (GSE38792).

Expression of IL-10 mRNA in subcutaneous fat tissue (GSE135917) and visceral adipose tissue (GSE38792) of OSAHS patients was not significantly different from that of the control group, as shown in Figure 5A and B.

## 4. Discussion

OSAHS is associated with many complications, specifically in patients with obesity.^[46]^ Moreover, previous studies have found inflammation and OSAHS to be closely related.^[47,48]^ Many complications of OSAHS are related to the induction of chronic inflammation, which is common in obesity and OSAHS. Many studies have investigated the potential relationship between the IL-10 levels and OSAHS; however, the results are contradictory. Therefore, the current meta-analysis can be used to answer the question focused on the association of IL-10 levels with the incidence of OSAHS.

Based on 31 studies in 25 articles, no variation was observed in the plasma/serum IL-10 level in patients with OSAHS and those in the control group. Bioinformatics analysis also revealed that there was no major variation in the IL-10 mRNA expression levels in subcutaneous adipose tissue and visceral adipose tissue between patients with OSAHS and the control group. IL-10 is secreted by activated T cells (mainly Treg cells, Th2 cells), lymphocytes, monocytes/macrophages, mast cells, etc. It is an anti-inflammatory factor that inhibits the pro-inflammatory factors TNF-α, IL-6, IL-8, etc.^[49]^ Leon-Cabrera et al^[12]^ showed that the severity of OSAHS was related to the concentration of anti-inflammatory factors, and the concentration of IL-10 decreased with the increase in Apnea–Hypopnea Index (AHI). However, there were only 29 OSAHS samples in the above experiment. A large sample cohort study (708 cases) conducted by Silva et al^[40]^ showed that CRP was the only inflammatory marker with a higher value in OSAHS patients than in patients without OSAHS, and no major variation was observed in the IL-10 expression levels of the 2 groups. In this study, after the combined analysis of a single small sample study, it was also found that there was no significant difference in the expression level of IL-10 between people with OSAHS and without OSAHS. This result was different from the results of some small sample studies. The reason could be their small sample size, and the conclusion might have some deviation. The purpose of meta-analysis was to combine and analyze the experiments of small samples to draw more reliable conclusions. Although OSAHS is an inflammatory disease with intermittent hypoxia, the degree of inflammatory reaction in patients may be relatively mild, and IL-10 expression level in blood and adipose tissue has not significantly decreased or increased. However, based on Chen VG’s study,^[25]^ IL-10 level in tonsils of children with OSAHS was higher, but the author also said that considering that IL-10 is an anti-inflammatory cytokine, tonsils did not seem to be a decisive factor in the pathophysiology of OSAHS inflammation.

Furthermore, there was a fact that children are still in the stage of growth and development; thus, their immune system has not been established and improved, and the immune response state is different from that of adults. However, IL-10 expression levels were also not associated with OSAHS when we performed subgroup analysis according to age, suggesting that age of participants did not affect the expression of IL-10. In addition, we also found that there was no significant difference between the patients with OSAHS and that of the control group in patients of Asian, Caucasian, and Latin American origin. Though race may affect the IL-10 levels in patients with OSAHS. Su et al^[41]^ found no difference in the gene frequency of IL-10-819 T/C between the OSAHS and control groups in a population from China; however, there was a difference in the gene frequency of IL-10-1082 G/A between the 2 groups. IL-10-1082 G/A and IL-10-1082 G/A may play an important protective role in the pathogenesis of OSAHS. However, it has not been reported that the polymorphism of IL-10 affects the expression of IL-10 protein. Perhaps changes in living environment, constitution, and diet of different races will affect people’s immune system, resulting in differences in IL-10, but these differences are relatively subtle. When multiple studies were combined and analyzed, the results showed that the relationship between the concentration of IL-10 and OSAHS was weak. When subgroup analysis was conducted according to the type of study design and disease severity, we did not find any variation in IL-10 expression levels between patients with OSAHS and the control group. It indicated that the type of study design and the severity of the disease did not affect the plasma/serum IL-10 level of OSAHS patients.

In addition, previous studies had confirmed that IL-10 levels negatively correlated with higher BMI scores.^[50,51]^ Leon Cabrera et al^[12]^demonstrated that the IL-10 levels in the serum of non-obese individuals decreased by 15% compared to that of obese individuals. Oxidative stress, validation, and sympathetic activation, which are known pathophysiological mechanisms in patients with OSA, also occur in obese patients; however, the correlation between the cytokine levels, OSA, and obesity is not clear. BMI may be a confounding factor in obese patients with OSA. Therefore, we conducted a subgroup analysis according to the average BMI. There was no major variation in plasma/serum IL-10 levels in patients with OSAHS and those in the control groups, whether BMI ≥ 30 or BMI < 30. According to our bioinformatics analysis, no major variation was observed in IL-10 levels in subcutaneous adipose tissue and visceral adipose tissue in patients with OSAHS and normal controls. These results suggested that BMI might not be the main factor affecting the expression of IL-10 in patients with OSAHS. Rajbhandari et al^[52]^ demonstrated that anti-inflammatory cytokine IL-10 improves insulin sensitivity, protects against diet-induced obesity, and elicits the browning of white adipose tissue. This finding is contradictory to the conclusion of our study. It is possible that our study included very few obese patients with OSAHS for evaluating the association between IL-10 concentration and OSAHS. Thus, additional studies with a larger number of obese patients are required to confirm the present results.

The exact mechanism of the relationship between IL-10 concentration and OSAHS are still unknown, but many hypotheses have been advanced. Repeated apnea at night in patients with OSA leads to hypoxia, and the resulting oxidative stress response can lead to systemic inflammation.^[53]^ In addition, nocturnal hypercapnia and sleep fragmentation in patients with OSA also results in the activation of pro-inflammatory pathways and the down-regulation of anti-inflammatory proteins. IL-10 is a pleiotropic cytokine mainly secreted by Th2 helper cells, which can widely inhibit the body’s inflammatory response. Gozal et al^[11]^ demonstrated that the circulating IL-10 levels in children with OSA was lower than that of the control group. The upper airway obstruction was relieved in following tonsillectomy in these patients, and the IL-10 levels returned to normal, indicating that airway stenosis during sleep could be related to hypoxia and interruption of IL-10 synthesis. As mentioned earlier, hypoxia has been shown to play a key role in the activation of the NF-κB pathway. In addition to the generation of IL-12 and TNF-α, it can also downregulate the synthesis of IL-10 in C57BL/6J mice.^[54]^ This noteworthy information indicated that the NF-κB-dependent signaling pathway plays an important role in the pathogenesis of OSA and suggests that NF-κB could be involved in the inhibition of IL-10. Some studies^[55,56]^ also considered hypoxia to inhibit the synthesis of IL-10; the imbalance between inflammatory cytokines and anti-inflammatory cytokines IL-10 can result in endothelial cell damage. However, most of the above studies had a small sample size, and the change in the degree of IL-10 was small, so the conclusion was different from that of this meta-analysis.

In general, the heterogeneity among meta-analysis studies is related to the quality, overall characteristics, experimental methods, and other factors. This study found that the relationship between IL-10 level and OSA was highly heterogeneous. In order to explore the source of heterogeneity, we conducted subgroup and meta-regression analyses. Meta-regression demonstrated that the P values of age, race, study design type, BMI, and AHI were greater than 0.05. In spite of the results of the meta-regression analysis, we further conducted subgroup analysis according to former factors. Unfortunately, no obvious sources of heterogeneity were found. Most participants of included studies were monitored using PSG I (polysomnography I), so no subgroup analysis was conducted according to the type of PSG; in the 31 studies, most of the experimental detection methods were ELISA, so this study did not conduct subgroup analysis according to the type of experimental detection methods. In addition, the sensitivity analysis demonstrated that no individual studies contributed to the high heterogeneity. Therefore, we speculate that several other factors result in the heterogeneity, including storage methods, experimental conditions, smoking status, lifestyle, environmental factors, and other potential confounding factors.

The meta-analysis results showed that the concentration of IL-10 might have little relationship with OSAHS. However, there are certain limitations to be considered while interpreting the results of the current meta-analysis. The most important limitation is that the evidence obtained was completely based on observational studies; hence, the potential confounding factors may not be controlled. Another important limitation is that the sample size included in most of the studies was small, which may not strongly prove the association between the IL-10 levels and OSAHS.

## 5. Conclusion

In conclusion, the current meta-analysis demonstrated that low IL-10 levels were not significantly associated with the risk of OSAHS. Well-designed large-scale studies involving patients of different populations and regions is strongly recommended.

## Acknowledgments

We thank Bullet Edits Limited for the linguistic editing and proofreading of the manuscript.

## Author contributions

**Conceptualization:** Xiaoyan Li, Qiuhua Sun.

**Data curation:** Xiaoyan Li, Qiuhua Sun.

**Formal analysis:** Xiaoyan Li, Lingyun Wu, Qiuhua Sun.

**Funding acquisition:** Xiaoyan Li, Qiuhua Sun.

**Investigation:** Xiaoyan Li, Qiuhua Sun.

**Methodology:** Xiaoyan Li, Qiuhua Sun.

**Project administration:** Xiaoyan Li, Qiuhua Sun.

**Resources:** Xiaoyan Li, Qiuhua Sun.

**Software:** Xiaoyan Li, Jie He, Qiuhua Sun.

**Supervision:** Xiaoyan Li, Qiuhua Sun.

**Validation:** Xiaoyan Li, Qiuhua Sun.

**Visualization:** Xiaoyan Li, Qiuhua Sun.

**Writing – original draft:** Xiaoyan Li, Lingyun Wu, Jie He.

**Writing – review & editing:** Xiaoyan Li, Lingyun Wu, Jie He.

## Supplementary Material


