# Supplementary material for: Interleukin-10 is not associated with obstructive sleep apnea hypopnea syndrome: A meta-analysis and meta-regression
Source: Medicine (Baltimore). 2023 Sep 22;102(38):e35036. doi: 10.1097/MD.0000000000035036 (PMC10519576; doi:10.1097/MD.0000000000035036)
Supplement: Supplementary file 1 [file medi-102-e35036-s001.docx]

Supplementary Table 1 Clinical and demographic characteristics of patients in two datasets.

|  | GSE135917 | | GSE38792 | |
| --- | --- | --- | --- | --- |
|  | Control (n = 8) | OSA(n=10) | Control (n = 8) | OSA(n=10) |
| Age (years) | 54.5±11.6 | 56.1±10.8 | 54.5±11.6 | 56.1±10.8 |
| Gender |  |  |  |  |
| Male | 1 | 3 | 1 | 3 |
| Female | 7 | 7 | 7 | 7 |
| Body mass index  (kg/m^2^) | 35.2±5.8 | 36.1±9.3 | 35.2±5.8 | 36.1±9.3 |
| RDI or AHI (events/hour) | 0.6±0.5 | 19.2±25.9 | 0.6±0.5 | 19.2±25.9 |
| Diabetes | 0 | 3 | 0 | 3 |
| Hypertension | 3 | 3 | 3 | 3 |
| Heart disease | 0 | 0 | 0 | 0 |
